# Obesity Reshapes the Microbial Population Structure along the Gut-Liver-Lung Axis in Mice

**DOI:** 10.3390/biomedicines10020494

**Published:** 2022-02-19

**Authors:** Apostolos Galaris, Dionysios Fanidis, Elli-Anna Stylianaki, Vaggelis Harokopos, Alexandra-Styliani Kalantzi, Panagiotis Moulos, Antigone S. Dimas, Pantelis Hatzis, Vassilis Aidinis

**Affiliations:** 1 Institute of Bioinnovation, Biomedical Sciences Research Center Alexander Fleming, 16672 Athens, Greece; galaris@fleming.gr (A.G.); fanidis@fleming.gr (D.F.); stylianaki@fleming.gr (E.-A.S.); kalatzi@fleming.gr (A.-S.K.); dimas@fleming.gr (A.S.D.); 2 Institute for Fundamental Biomedical Research, Biomedical Sciences Research Center Alexander Fleming, 16672 Athens, Greece; harokopos@fleming.gr (V.H.); moulos@fleming.gr (P.M.); hatzis@fleming.gr (P.H.)

**Keywords:** obesity, high-fat diet, microbiome, 16S rRNA, gut, liver, lung, comparative analysis, firmicutes, staphylococcus

## Abstract

The microbiome is emerging as a major player in tissue homeostasis in health and disease. Gut microbiome dysbiosis correlates with several autoimmune and metabolic diseases, while high-fat diets and ensuing obesity are known to affect the complexity and diversity of the microbiome, thus modulating pathophysiology. Moreover, the existence of a gut-liver microbial axis has been proposed, which may extend to the lung. In this context, we systematically compared the microbiomes of the gut, liver, and lung of mice fed a high-fat diet to those of littermates fed a matched control diet. We carried out deep sequencing of seven hypervariable regions of the 16S rRNA microbial gene to examine microbial diversity in the tissues of interest. Comparison of the local microbiomes indicated that lung tissue has the least diverse microbiome under healthy conditions, while microbial diversity in the healthy liver clustered closer to the gut. Obesity increased microbial complexity in all three tissues, with lung microbial diversity being the most modified. Obesity promoted the expansion of Firmicutes along the gut-liver-lung axis, highlighting staphylococcus as a possible pathologic link between obesity and systemic pathophysiology, especially in the lungs.

## 1. Introduction

The microbiome, the sum of commensal, symbiotic, and pathogenic organisms that populate animal bodies, is increasingly recognized as a major player in tissue homeostasis in health and disease [1], modulating a variety of host functions, including immunity and inflammation [2], as well as energy homeostasis and metabolism [3]. Changes in microbial population structure and the ensuing local or systemic effects can be induced by different environmental factors, most notably exposure to antibiotics and dietary changes, while the efficacy of various medications has been suggested to correlate with microbiome perturbations and vice versa [1].

Most microorganisms reside within the intestine. It is well established that the gut microbiome participates in multiple homeostatic functions essential for the host, including nutrient absorption and education of the immune system. Alterations in the composition and complexity of microbiomes can harm the health of an organism. Such alterations lead to dysbiosis and have been associated with autoimmune and metabolic diseases, mostly via secreted microbial metabolites [4]. Non-alcoholic fatty liver disease (NAFLD) has been linked to dysbiosis [5,6], highlighting a connection between gut microbiota and the liver, referred to as the gut-liver axis [7]. The gut and liver are in direct contact through the biliary tract and the portal vein, and disturbances in gut barrier functions result in an increased influx of bacteria and their metabolites to the liver [7]. Moreover, a gut-brain microbial axis has also been proposed [8], suggesting that the gut microbiome may also affect distant organs. In the same vein, a gut-lung axis has also been proposed, and dysbiosis has been associated most notably with asthma and chronic obstructive pulmonary disease (COPD) [9]. It is noteworthy that elder individuals become more susceptible to these respiratory diseases, while the process of aging has also been associated with microbial dysbiosis [10]. Moreover, the lungs—believed until recently to be sterile—have been found to contain their own bacterial flora, which is deregulated in disease states [11]. Specific bacterial species have recently been associated with disease status in COPD patients [12] and with mortality in idiopathic pulmonary fibrosis (IPF) patients [13]. Liver functions, including endotoxin and bacterial clearance, have been suggested as critical determinants of lung pathophysiology in acute respiratory distress syndrome (ARDS) [14], while the contribution of a liver-lung axis has also been proposed in alcohol-induced liver diseases [15]. Moreover, a fiber-rich diet has been shown to confer reduced risk for COPD, possibly through metabolic, liver-mediated effects on innate immunity. This points to a potential gut-liver-lung axis [16] that may further involve metabolic regulation of microbial dysbiosis. However, the gut-liver-lung axis is thought to be imposed mainly via microbial metabolites. In that respect, the actual microbial populations of the different organs along with the full gamut of metabolites and their possible similarities or differences have not been fully elucidated.

Obesity and obesity-related metabolic disorders are linked to lipid biosynthetic pathways in the liver and have been associated with the composition of the human gut microbiome [4,17]. To date, multiple studies are starting to highlight the important contribution of the microbiome to human health, but there remains a notable heterogeneity in published results. Moreover, much less is known about the effects of obesity on the microbial composition of the liver and lungs or along the gut-liver-lung axis. In this context, we examined the bacterial composition along the gut-liver-lung axis upon high-fat diet (HFD)-induced obesity in wild type (wt) C57Bl6 mice, based on 16 rRNA gene (V2-4, V6-9) sequencing.

## 2. Materials and Methods

### 2.1. Animals

All mice (four per dietary regime) were bred under specific-pathogen-free (SPF) conditions at the local animal facility at 20–22 °C, 55 ± 5% humidity, and a 12-h light/dark cycle; water and food were provided ad libitum. All experiments on mice were in line with ARRIVE guidelines and were approved by the Veterinary Service and Fishery Department of the local governmental prefecture (#740336), following the positive opinion of the Institutional Protocol Evaluation Committee BSRC Alexander Fleming. Experimental animals were on an HFD (60% fat VHFD, D12492i, Research diets, New Brunswick, New Jersey, USA), and their littermate control animals were fed the corresponding matched control diet (CD) (10% fat, D12450J, Research diets, New Brunswick, New Jersey, USA) for 16 weeks starting after their ablactation. All mice were co-housed continuously throughout the experiment, taking all necessary precautions for cross-contamination.

### 2.2. Tissue Sampling

Mice were anesthetized using a xylazine/ketamine/atropine mixture (10 mg/100 mg/0.05 mg/kg body weight respectively) and then euthanized by a gradual supply of carbon dioxide. Small intestines were isolated; the first ~1 cm right after stomach was discarded, and samples of ~50 mg were collected and immediately transferred into liquid nitrogen. No washing of the intestine was performed. Next, perfusion with PBS was performed, as previously described [18], and liver and lung samples were isolated and immediately transferred into liquid nitrogen. All tissue samples were stored at −80 °C until processing.

### 2.3. Histopathology

Right lung tissues and medial liver lobes were fixed using 10% neutral buffered formalin. The gut was fixed using a fixation buffer containing 50% Ethanol and 5% acetic acid. All tissues were placed in paraffin. 4 μm sections were prepared and stained with hematoxylin/eosin (H&E) standard protocols. Tissue imaging was performed using a Nikon Eclipse E800 microscope (Nikon Corp., Shinagawa-ku, Japan) attached to a Q Imaging EXI Aqua digital camera, using the Q-Capture Pro software (v7.0, QImaging, Surrey, BC, Canada).

### 2.4. Plasma Sampling

Plasma was collected from every subject as previously described (Barbayianni, Ninou, et al. 2018). In brief, after euthanasia of the animals, the blood was collected through the inferior vena cava, and EDTA was added to a final concentration of 10%. The samples were then centrifuged for 20 min at 2000 *g* at 4 °C. Plasma was transferred and stored at 4 °C until biochemical analysis was performed using a Beckman Coulter AU480 Clinical Chemistry Analyzer based at the BSRC ‘Alexander Fleming’ phenotyping facility for the estimation of Alanine Transaminase (ALT) (OSR6107, Beckman Coulter, Brea, CA, USA) and Aspartate Transaminase (AST) (OSR6109, Beckman Coulter, Brea, CA, USA) levels.

### 2.5. DNA Extraction and 16s Library Preparation

Total genomic DNA was extracted from approximately 50 mg of tissue using the DNeasy^®^ Blood & Tissue Kit (Cat. Nos. 69504 and 69506, Qiagen, Hilden, Germany) following the manufacturer’s suggested protocol. 2-8 μL (~100–250 ng) of genomic DNA from each sample was used with the Ion 16S™ Metagenomics Kit (A26216, ThermoFisher Scientific, Waltham, MA, USA) to amplify the 16S hypervariable regions. The kit used includes two sets of primers targeting V2, V4, V8— And V3, V6-7, and V9 regions, respectively. After sample purification, DNA libraries were prepared with the Ion Plus Fragment Library Kit (4471252, ThermoFisher Scientific, Waltham, MA, USA) according to the manufacturer’s instructions. The libraries were pooled and sequenced on an Ion Proton™ System. Briefly, the genomic DNA from each sample was divided into two parts, and each was amplified with a different pool of primers needed to cover the 16S hypervariable regions. Given that distinct hypervariable regions may contribute in varying degrees to the identification of bacterial species, especially for low-level taxa [19], we amplified seven of nine hypervariable regions (V2-4, V6-9), thus enabling broad-range identification of bacterial populations. Following amplification, the two reactions were pooled, and all samples were further purified with Agencourt AMPure XP Beads (A63881, Beckman Coulter, Brea, CA, USA). Sample concentration was measured using the Qubit dsDNA HS Assay Kit (Q32851 ThermoFisher Scientific, Waltham, MA, USA), and approximately 50 ng of amplified DNA was used to prepare DNA libraries with the Ion Plus Fragment Library Kit (4471252, ThermoFisher Scientific, Waltham, MA, USA). End repair was followed by adaptor ligation and nick repair. After purification, libraries were amplified and further purified. Each library’s quality and quantity was assessed on a Bioanalyzer using the DNA High Sensitivity Kit reagents and protocol (5067-4626, Agilent Technologies, Santa Clara, CA, USA). Quantified libraries were pooled to a final concentration of 7 pM. The pools were then processed, templated, and enriched on an Ion Proton One Touch system. Templating was performed using the Ion PI™ Hi-Q™ OT2 200 Kit (A26433, ThermoFisher Scientific, Waltham, MA, USA), and sequencing was performed using the Ion PI™ Hi-Q™ Sequencing 200 Kit on Ion Proton PI™ V3 chips (A26771, ThermoFisher Scientific, Waltham, MA, USA) according to commercially available protocols. The Ion Proton™ System [20] was used for high-throughput sequencing according to the manufacturer’s instructions. The above process is illustrated in Figure S1A.

### 2.6. 16S rRNA Gene Sequencing Data Analysis

Fastq files were quality controlled using DADA2 functions [21] in order to trim 14b read left ends, as suggested for IonTorrent data. Reads under 50 bp were subsequently removed along with those sequences with more than four expected errors. Trimmed reads were aligned against the human and mouse genomes using the FastQ Screen tool (v0.14.01) [22] to detect contaminant sequences. No microbial reads were filtered from the initial raw fastq files, which were pooled per sampled tissue and diet prior to quality control. Filtration and trimming were performed as before, prior to denoising and chimera removal using DADA2 (Figure S2; Table S1). Processed files were assigned to amplicon sequence variants (ASVs). Taxonomy assignment was performed using the SILVA database [23], followed by gene copy number (GCN) normalization. For GCN correction, abundance values were divided by each taxon’s 16S gene copy number as recorded in rrnDB (v5.7 NCBI) [24] using an in-house script. Taxa with no records in rrnDB were not considered for downstream analysis. The above process is depicted in Figure S1B.

### 2.7. Statistical Analysis

Statistical analysis was performed using the Prism 6 software (GraphPad, San Diego, CA, USA), as specifically indicated in the text and the corresponding figure legends.

## 3. Results

### 3.1. High Fat Diet Induces Obesity and NAFLD

To examine the effect of obesity on the microbiome and possible interconnections of the gut-liver axis with the lung, we fed wt C57Bl6 mice with a non-toxic HFD for 16 weeks, starting right after their ablactation, at four weeks of age. Littermate mice fed a matched CD were used as controls.

**Figure 1:**
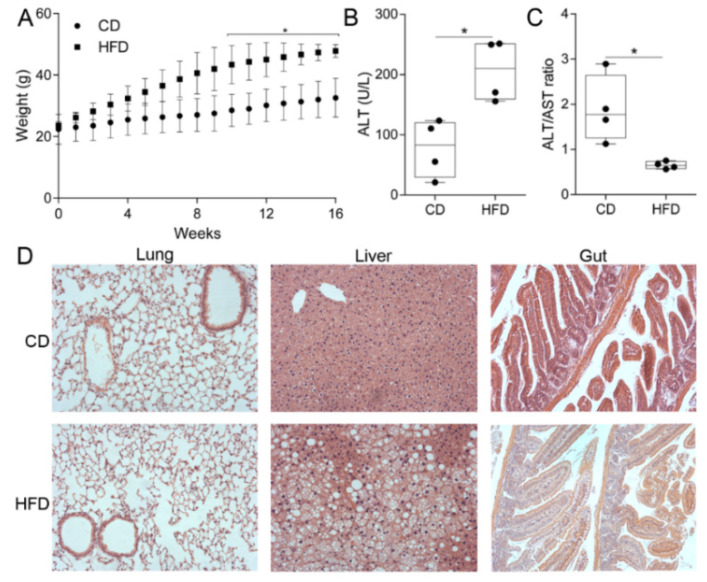
Mice fed with a high-fat diet (HFD) developed obesity and non-alcoholic liver fatty liver disease (NAFLD). Mice fed with HFD for 16 weeks presented with (**A**) statistically significant higher body weight from the tenth week of HFD onwards, (**B**) elevated ALT levels, and (**C**) decreased AST/ALT ratio in plasma after 16 weeks of HFD. (**D**) Representative images from the histopathology (hematoxylin & eosin staining) of gut, liver, and lung tissues, illustrating lipid deposition in the liver and gut after 16 weeks of HFD. Hematoxylin stains cell nuclei (purple) and eosin stains the extracellulal matrix (pink). The “bubbles” appearing in HDF liver and gut samples are lipid droplets. Statistical significance was assessed through the Friedman test followed by pairwise Mann-Whitney tests (in **A**) and Mann-Whitney tests (in **B** and **C**); * *p* < 0.05 was considered significant.

As expected, mice fed with HFD gained more weight than their littermate controls (Figure 1A). Obese mice presented with elevated Alanine Transaminase (ALT) levels in their plasma (Figure 1B) and decreased AST/ALT (AST: Aspartate Transaminase) ratios (Figure 1C), indicating liver damage as well as lipid deposition in the liver (Figure 1D), both reminiscent of NAFLD. Lipid deposition was also observed in the gut but not in the lung (Figure 1D).

### 3.2. Obesity Increases Microbial Complexity along the Gut-Liver-Lung Axis

Quality controlled, denoised fastq files were pooled per tissue and diet and were assigned to amplicon sequence variants (ASVs). Following abundance level correction for differing 16S gene copy numbers between taxa (GCN correction), we detected in total 29 phyla, 59 classes, 130 orders, 227 families, and 585 species (Figure S3A).

**Figure 2:**
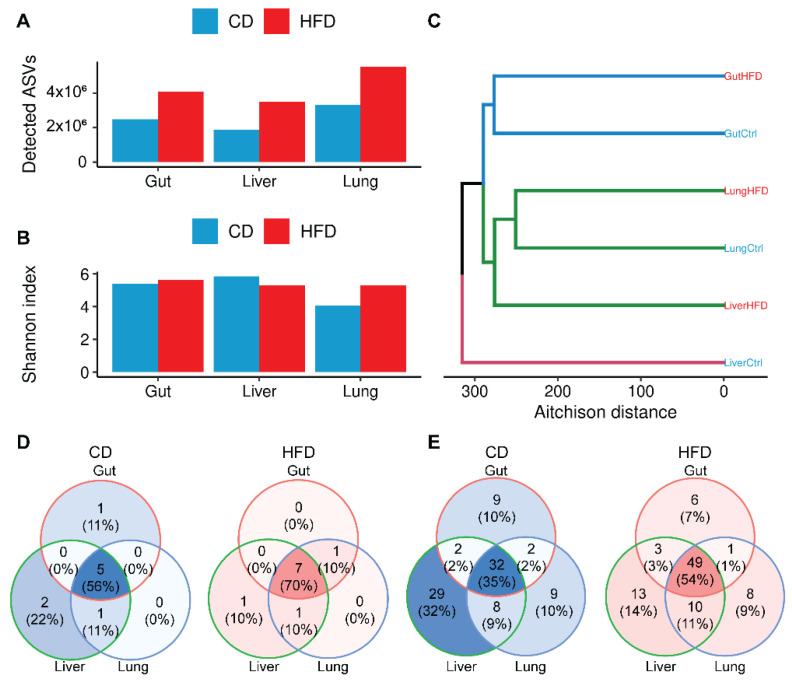
HFD-driven obesity triggers changes in the microbiome composition of all tissues examined. (**A**) HFD-driven obesity results in a greater number of detected Amplicon Sequence Variants (ASVs) in all tissues. (**B**) α-diversity per sample and diet. Shannon’s index was used to evaluate sample biodiversity per tissue and diet. (**C**) Similarity of samples per tissue and diet as described by β-diversity. Aitchison distance was used to account for the compositional nature of 16S rRNA sequencing data. (**D**) Venn diagrams of common phyla or (**E**) families upon CD or HFD in gut, liver, and lung.

To reveal patterns of microbiota composition in each gut, liver, and lung, we calculated bacterial diversity in terms of microbial richness (observed number of ASVs) and biodiversity (Shannon’s index). No differences were observed in host tissue 18S rRNA gene levels detected by RT-PCR and used as a negative loading control (Figure S3B). As shown in Figure 2A, obesity resulted in higher microbial richness compared to equivalent controls in all three tissues studied, an observation consistent with the higher numbers of microbial taxa assigned upon HFD (Figure S3C). We report that although the lung is the tissue with the least diverse microbiome under healthy conditions, its microbial diversity is affected the most by HFD-driven obesity and reaches levels similar to those recorded for the other two tissues under these conditions (Figure 2B).

To identify overall similarities in taxonomic composition between tissues (β-diversity), we calculated the Aitchison distance of tissue microbiomes under healthy and obese conditions. This distance metric was selected in order to take into consideration the compositional nature of 16S rRNA gene sequencing data [19]. As shown in Figure 2C, we observe that HFD-driven obesity shifts the composition of the lung microbiome closer to that of the liver. Moreover, HFD-driven obesity results in a relative increase in phyla and families that are shared across the examined tissues (Figure 2D,E; Tables S2 and S3).

### 3.3. Obesity Favors Firmicutes and, Most Notably, Staphylococcus Expansion in All Tissues

**Figure 3:**
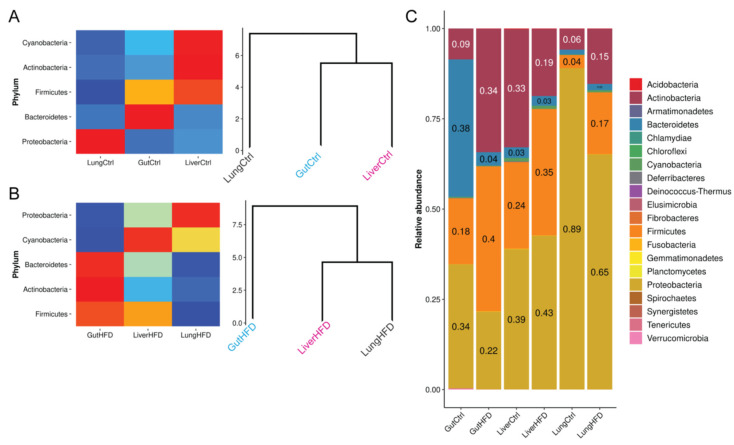
Relative abundance of phyla detected in gut, liver, and lung of control and HFD-fed mice. Heatmap and respective dendrogram of non-zero abundance phyla under CD (**A**) and HFD (**B**). (**C**) Relative abundance of all detected phyla. Relative abundance calculations for all panels were based on GCN values. For panels A and B, only phyla with an abundance greater than zero after value rounding to two decimal places were considered. Heatmaps are scaled per phylum. Manhattan distance was used to perform hierarchical clustering of the tissues with complete linkage in panels A and B.

Given detected phyla, our data indicate that gut, liver, and lung share the same top-level taxa: Proteobacteria, Actinobacteria, Bacteroidetes, Cyanobacteria, and Firmicutes (Figure 3). Under CD and based on relative abundance, the gut microbiome is more closely related to that of the liver (Figure 3A). Upon HFD administration, this balance is disturbed, and the liver microbiome shifts closer to the lung (Figure 3B). This is also indicated by the recorded β-diversity (Figure 2C) and because more taxa are shared across the liver and lung at different taxonomic levels (Figure 2D,E).

In CD liver and lung tissue, we report that Proteobacteria are the most abundant bacteria, followed by Actinobacteria and Firmicutes (Figure 3C). This composition is slightly different in the CD gut, with the most abundant Bacteroidetes being followed by Proteobacteria and Firmicutes. Upon HFD, liver and lung are occupied, in order of abundance, by Proteobacteria, Firmicutes, and Actinobacteria. On the other hand, the gut is mainly populated by Firmicutes and Actinobacteria, followed by Proteobacteria. Notably, our findings show that HFD-driven obesity favors expansion of Firmicutes in all three tissues studied.

**Figure 4:**
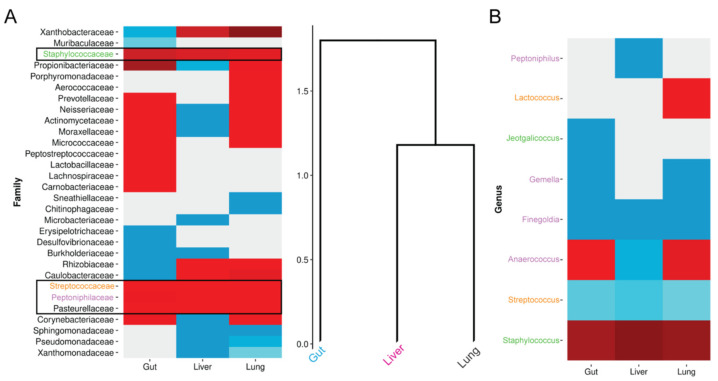
HFD consistently affects specific microbial families and genera. (**A**) Heatmap and tissue dendrogram of HFD-to-CD differences in family relative abundance (**B**) Heatmap of HFD-to-CD difference in genera relative abundance. For both panels, only those taxa with HFD-to-CD relative abundance differences other than zero in at least one tissue after rounding to two decimal places were considered. Heatmaps are scaled per taxon. Manhattan distance was used to perform hierarchical clustering of tissues with complete linkage. Relative abundance was calculated based on GCN abundance values. Families and respective genera are coded with the same color in both panels.

Deeper examination at the taxonomic level revealed that the Firmicutes families that expanded under HFD-driven obesity in all tissues examined were Staphylococcaceae, Streptococcaceae, and Peptoniphilaceae (Figure 4A); Pasteurelacceae family (Proteobacteria) expanded as well. Concerning detected genera, two, three, and eleven genera were detected within the Streptococcaceae, Staphylococcaceae, and Peptoniphilaceae taxa, respectively (Table S4). Among them, and under HFD-driven obesity, Staphylococcus has an increasing tendency in all three tissues relative to CD conditions (Figure 4B).

A notable point that we raise given our findings is that the three genera most consistently affected by HFD (Staphylococcus, Streptococcus, and Finegoldia) have been shown to produce superantigens (SAg), potent immunomodulators that are produced by microorganisms (Figure 4B). Given that SAgs are produced by specific, mostly bacterial species, we searched UniProt to track them in our dataset. A search of the database with the UniProt keyword ‘Superantigen’ revealed several UniProt-KB and Uni-Prot-TrEMBL entries, which, when intersected with those of our 16S amplicon sequencing, led to the detection of six SAg-related species (Table S5), of which four belong to genera whose diversity was affected upon administration of HFD in at least one of tissue. This points to the existence of a potential bacterial-driven pathogenic mechanism, which should be further explored.

## 4. Discussion

In this report, we examined the microbial composition of the guts, livers, and lungs of mice fed a high-fat diet (HFD) compared to littermates fed a matched control diet (CD). Towards this goal, we employed sequencing of seven (out of nine) 16S rRNA gene hypervariable regions. Obesity was shown to increase microbial complexity along the gut-liver-lung axis, promoting the expansion of Firmicutes, especially of Staphylococcus.

To induce obesity in mice, we utilized a non-toxic (not methionine- or choline-deficient) HFD containing 20% protein, 20% kcal fat, and 60% kcal carbohydrate with a total of 20% kcal energy density (5.21 kcal/g). As a result, the mice gained weight (unlike with toxic diets), with lipids being deposited in both liver and gut, but not in lung tissue (Figure 1).

The technique of 16S rRNA gene amplicon sequencing has revolutionized microbiomics [25]. Although most reported sequencing protocols examine one or two hypervariable regions [25], we selected seven hot spots (V2-V4 and V6-9) for amplification, given that each of these nine sub-regions has a distinct potential for distinguishing between microbial taxa, especially for lower taxonomic levels [19]. This selection also serves to avoid skewing of taxa distributions, as, for example, V1-V2 present poor performance in assigning sequences to the phylum of Proteobacteria [26], which are underrepresented in most studies. Additionally, amplicon sequence variants (ASVs) were preferred to operational taxonomic units (OTUs), given that OTUs represent certain bacterial taxa inadequately [27] and can systematically polarize diversity metrics due to reference incompleteness [28]. Furthermore, we performed GCN correction to normalize microbial abundance levels for copy numbers.

In healthy CD mice, we report that all three tissues examined (gut, liver, and lung) were populated chiefly by four phyla: Proteobacteria, Actinobacteria, Bacteroidetes, and Firmicutes (Figure 3C), as previously reported for the gut in mice [29,30] and humans [31,32]. Interestingly, Cyanobacteria were also detected in all three tissues consistent with previous observations of the phylum in mouse lung [33], lower respiratory tract [34], and gastrointestinal canal [35]. Gut microbiome diversity is known to increase with age. In humans, it stabilizes at the age of three and is largely dominated by Bacteroidetes, Firmicutes, and Proteobacteria [36,37]. The liver is the first organ to encounter gut-derived bacteria upon intestinal barrier dysfunction through the portal vein, as well as bacteria from systemic infections via the hepatic artery, which are then removed from circulation through hepatic filtering [38,39]. Bacterial clearance by the liver has been suggested to occur in a dual-track mode: rapidly via Kupffer cell scavenger receptors and through a slower process involving different immune system mechanisms, making the translocated microorganisms available for the induction of adaptive immunity [38,39]. Given the above mechanisms, it is not surprising that bacterial rDNA was detected, to the best of our knowledge for the first time, in the healthy livers of mice (Figure 2, Figure 3 and Figure 4). Our findings likely portray a snapshot of the process of bacterial clearance by the liver or remnants of degraded bacteria. Microbial diversity in the healthy liver clustered closer to that of the gut ( C and Figure 4A), pointing to a larger contribution of portal vein-derived gut-leaked microbes than previously thought. This mechanism has been proposed to contribute to the worsening of human liver diseases [40]. Importantly, bacterial rDNA was recently detected in the liver of healthy human individuals [41]. Concerning lung tissue, which until recently was believed to be sterile, studies in mice have reported colonization with Proteobacteria, Firmicutes, and Bacteroidetes [42]. These phyla are also reported to be the most prevalent in the human adult lung [43,44]. We report that the mouse lung microbiome was dominated mainly by Proteobacteria, Firmicutes, Bacteroidetes, and Actinobacteria under both CD and HFD (Figure 3C), although α-diversity levels showed that the lung was the organ/tissue with the least diverse microbiome (Figure 2B).

Obesity is a complex disease characterized by extensive lipid deposition throughout the body and increases the risk for multiple diseases [45,46]. The gut microbiome contributes to the pathophysiology of obesity [47], and the ‘obese’ microbiome has been suggested to harvest energy from nutrients with increased capacity [48]. Gut microbiota have been shown to regulate body fat content in mice [11] and are regarded as a putative target for obesity treatments [47]. As shown in the present study, HFD-driven obesity suppresses Bacteroidetes in the gut of mice (Figure 3C), an effect also observed in aging mice [49]. Moreover, HFD-driven obesity was found to increase the Firmicutes to Bacteroidetes ratio in the gut (Figure 3C), expanding on previous studies in genetically engineered obese mice and obese humans [50,51]. An increase in the diversity of Firmicutes upon HFD-driven obesity was also observed, for the very first time, in the liver (Figure 3C). This observation may reflect gut microbiome leaking in the circulation and being cleared in the liver. However, a similar increase in Firmicutes was also recorded for lung tissue (Figure 3C), supporting the view that obesity stimulates systemic changes in microbiome composition, but also the existence of a gut-liver-lung axis. Such an inter-organ network maybe exist through different communication mechanisms that have been proposed in the past, including commensal microbe translocation [52] and chemical communication through bacterial metabolites (e.g., SCFAs) and tissue products [52,53].

Among Firmicutes, an abundance of Staphylococcus was characterized by an increasing tendency in all three tissues upon HFD-driven obesity (Figure 4). Staphylococcus is a Gram-positive, opportunistic pathogen that colonizes the skin as well as mucosal surfaces and may cause a range of infections in healthy and immunocompromised individuals, as well as in recovering postoperative patients. Obesity has been suggested to increase the risk of Staphylococcus colonization in humans, with obese individuals being more susceptible to pneumonia, wound infections, bacteremia, and sepsis [54]. Increased abundance of Staphylococcus and Staphylococcaceae family members have also been reported in asthma [44], where obesity is a major risk factor and a disease modifier in children and adults [55]. The gut microbiome has been suggested to link obesity to asthma. Staphylococcus is a likely component of this link, with increased lung colonization by this microbe upon obesity. In cystic fibrosis (CF), *Staphylococcus aureus* is the second most commonly isolated pathogen from the airways of patients. The increasing prevalence of obesity in CF patients is associated with further impairment of lung function [56]. Lung Staphylococcus burden has also been found to be increased in chronic hypersensitivity pneumonitis (CHP) [57], has been associated with progression of idiopathic pulmonary fibrosis (IPF) [58], and a Staphylococcus pro-apoptotic peptide has been correlated with acute exacerbations of IPF [59]. Therefore, as the gut microbiome has been linked with that of the liver [40] and the lung [55] in different disease contexts and obesity, Staphylococcus may be a potential pathogenic link between the three organs under divergence from a steady state.

Furthermore, HFD-induced obesity was shown to affect bacterial species, mainly Staphylococcus, capable of producing superantigens (SAgs; Table S5), which are potent immunostimulatory molecules [60]. Chronic exposure to *S. aureus* SAg toxic shock syndrome toxin-1 (TSST-1) has been shown to facilitate the development of diabetic complications in rabbits [61], while TSST-1 has also been shown to stimulate cytokine production from adipocytes [62], thus possibly contributing to the low-grade systemic inflammation associated with obesity and diabetes. Moreover, staphylococcal SAg enterotoxin B (SEB) was shown to cause interstitial pneumonia in both autoimmune and non-autoimmune mice [63], although obesity is associated with decreased pneumonia risk and mortality, reflecting the ‘obesity paradox’.

The presented study is characterized by certain limitations. First, the pooled sample analysis design followed limits our results to a mostly descriptive nature. In addition, 16S rRNA gene amplicon sequencing data cannot be used directly for functional annotation. This, in combination with the study design, does not enable a deeper examination of proposed pathogenic mechanisms.

In conclusion, in addition to lipid deposition throughout the body and the triggering of NAFLD, obesity was shown to increase microbial complexity along the gut-liver-lung axis and, as a result, to possibly predispose mice to a series of metabolic diseases via increased abundance in Staphylococcus and other species.

## Data Availability

Processed data presented in this study are openly available in Zenodo at DOI: https://doi.org/10.5281/zenodo.6141436.

## Supplementary Materials

The following supporting information can be downloaded at: https://www.mdpi.com/article/10.3390/biomedicines10020494/s1, Figure S1: Schematic overview of experimental design and 16S rDNA sequencing and data analysis. Figure S2: Data quality control and error rate estimation; Figure S3: Obesity increased microbial complexity along the gut-liver-lung axis. Table S1: Quality control metrics before (grey-colored cells) and after (white-colored cells) human and bacteria sequences removal; Table S2. Important taxa as presented in Figure 2D,E.; Table S3: Number of shared taxa between any pairwise combination of experimental conditions; Table S4: Detected genera belonging to the Streptococcaceae, Staphylococcaceae, Peptoniphilaceae, and Pasteurelacceae families. Red marked are those affected by a high-fat diet in at least one tissue as defined by HFD-to-control relative abundance difference (non-zero difference); Table S5: Detected species related with superantigen proteins as recorded by UniProt database and/or respective literature.

## References

[1] Cho I., Blaser M.J. (2012). The human microbiome: At the interface of health and disease. Nat. Rev. Genet..

[2] Belkaid Y., Hand T.W. (2014). Role of the Microbiota in Immunity and Inflammation. Cell.

[3] Cani P.D., Van Hul M., Lefort C., Depommier C., Rastelli M., Everard A. (2019). Microbial regulation of organismal energy homeostasis. Nat. Metab..

[4] Fan Y., Pedersen O. (2021). Gut microbiota in human metabolic health and disease. Nat. Rev. Microbiol..

[5] Jiang W., Wu N., Wang X., Chi Y., Zhang Y., Qiu X., Hu Y., Li J., Liu Y. (2015). Dysbiosis gut microbiota associated with inflammation and impaired mucosal immune function in intestine of humans with non-alcoholic fatty liver disease. Sci. Rep..

[6] Zhu L., Baker S.S., Gill C., Liu W., Alkhouri R., Baker R.D., Gill S.R. (2013). Characterization of gut microbiomes in nonalcoholic steatohepatitis (NASH) patients: A connection between endogenous alcohol and NASH. Hepatology.

[7] Tripathi A., Debelius J., Brenner D.A., Karin M., Loomba R., Schnabl B., Knight R. (2018). The gut–liver axis and the intersection with the microbiome. Nat. Rev. Gastroenterol. Hepatol..

[8] Morais L.H., Schreiber H.L., Mazmanian S.K. (2021). The gut microbiota–brain axis in behaviour and brain disorders. Nat. Rev. Microbiol..

[9] Zhang D., Li S., Wang N., Tan H.-Y., Zhang Z., Feng Y. (2020). The Cross-Talk Between Gut Microbiota and Lungs in Common Lung Diseases. Front. Microbiol..

[10] Saint-Criq V., Lugo-Villarino G., Thomas M. (2021). Dysbiosis, malnutrition and enhanced gut-lung axis contribute to age-related respiratory diseases. Ageing Res. Rev..

[11] Bäckhed F., Ding H., Wang T., Hooper L.V., Koh G.Y., Nagy A., Semenkovich C.F., Gordon J.I. (2004). The gut microbiota as an environmental factor that regulates fat storage. Proc. Natl. Acad. Sci. USA.

[12] Bowerman K.L., Rehman S.F., Vaughan A., Lachner N., Budden K.F., Kim R.Y., Wood D.L.A., Gellatly S.L., Shukla S.D., Wood L.G. (2020). Disease-associated gut microbiome and metabolome changes in patients with chronic obstructive pulmonary disease. Nat. Commun..

[13] Mackintosh J.A., Desai S.R., Adamali H., Patel K., Chua F., Devaraj A., Kouranos V., Kokosi M., Margaritopoulos G., Renzoni E. (2019). In patients with idiopathic pulmonary fibrosis the presence of hiatus hernia is associated with disease progression and mortality. Eur. Respir. J..

[14] Herrero R., Sánchez G., Asensio I., López E., Ferruelo A., Vaquero J., Moreno L., de Lorenzo A., Bañares R., Lorente J.A. (2020). Liver–lung interactions in acute respiratory distress syndrome. Intensiv. Care Med. Exp..

[15] Arteel G.E. (2020). Liver-lung axes in alcohol-related liver disease. Clin. Mol. Hepatol..

[16] Young R.P., Hopkins R., Marsland B.J. (2016). The Gut–Liver–Lung Axis. Modulation of the Innate Immune Response and Its Possible Role in Chronic Obstructive Pulmonary Disease. Am. J. Respir. Cell Mol. Biol..

[17] Stanislawski M.A., Dabelea D., Lange L.A., Wagner B.D., Lozupone C.A. (2019). Gut microbiota phenotypes of obesity. Biofilms Microbiomes.

[18] Barbayianni I., Ninou I., Tzouvelekis A., Aidinis V. (2018). Bleomycin Revisited: A Direct Comparison of the Intratracheal Micro-Spraying and the Oropharyngeal Aspiration Routes of Bleomycin Administration in Mice. Front. Med..

[19] Gloor G.B., Macklaim J.M., Pawlowsky-Glahn V., Egozcue J.J. (2017). Microbiome Datasets Are Compositional: And This Is Not Optional. Front. Microbiol..

[20] Rothberg J.M., Hinz W., Rearick T.M., Schultz J., Mileski W., Davey M., Leamon J.H., Johnson K., Milgrew M.J., Edwards M. (2011). An integrated semiconductor device enabling non-optical genome sequencing. Nature.

[21] Callahan B.J., Mcmurdie P.J., Rosen M.J., Han A.W., Johnson A.J.A., Holmes S.P. (2016). DADA_2_: High-resolution sample inference from Illumina amplicon data. Nat. Methods.

[22] Wingett S., Andrews S. (2018). FastQ Screen: A tool for multi-genome mapping and quality control. F1000Research.

[23] Quast C., Pruesse E., Yilmaz P., Gerken J., Schweer T., Yarza P., Peplies J., Glöckner F.O. (2013). The SILVA ribosomal RNA gene database project: Improved data processing and web-based tools. Nucleic Acids Res..

[24] Stoddard S.F., Smith B.J., Hein R., Roller B.R., Schmidt T.M. (2015). rrnDB: Improved tools for interpreting rRNA gene abundance in bacteria and archaea and a new foundation for future development. Nucleic Acids Res..

[25] Bharti R., Grimm D.G. (2021). Current challenges and best-practice protocols for microbiome analysis. Briefings Bioinform..

[26] Johnson J.S., Spakowicz D.J., Hong B.-Y., Petersen L.M., Demkowicz P., Chen L., Leopold S.R., Hanson B.M., Agresta H.O., Gerstein M. (2019). Evaluation of 16S rRNA gene sequencing for species and strain-level microbiome analysis. Nat. Commun..

[27] Větrovský T., Baldrian P. (2013). The Variability of the 16S rRNA Gene in Bacterial Genomes and Its Consequences for Bacterial Community Analyses. PLoS ONE.

[28] Callahan B.J., McMurdie P.J., Holmes S.P. (2017). Exact sequence variants should replace operational taxonomic units in marker-gene data analysis. ISME J..

[29] Liu C., Zhou N., Du M.-X., Sun Y.-T., Wang K., Wang Y.-J., Li D.-H., Yu H.-Y., Song Y., Bai B.-B. (2020). The Mouse Gut Microbial Biobank expands the coverage of cultured bacteria. Nat. Commun..

[30] Lagkouvardos I., Pukall R., Abt B., Foesel B.U., Meier-Kolthoff J.P., Kumar N., Bresciani A., Martínez I., Just S., Ziegler C. (2016). Erratum: Corrigendum: The Mouse Intestinal Bacterial Collection (miBC) provides host-specific insight into cultured diversity and functional potential of the gut microbiota. Nat. Microbiol..

[31] Mahowald M.A., Rey F.E., Seedorf H., Turnbaugh P.J., Fulton R.S., Wollam A., Shah N., Wang C., Magrini V., Wilson R.K. (2009). Characterizing a model human gut microbiota composed of members of its two dominant bacterial phyla. Proc. Natl. Acad. Sci. USA.

[32] Turnbaugh P.J., Hamady M., Yatsunenko T., Cantarel B.L., Duncan A., Ley R.E., Sogin M.L., Jones W.J., Roe B.A., Affourtit J.P. (2009). A core gut microbiome in obese and lean twins. Nature.

[33] Barfod K.K., Roggenbuck M., Hansen L.H., Schjørring S., Larsen S.T., Sørensen S.J., Krogfelt K.A. (2013). The murine lung microbiome in relation to the intestinal and vaginal bacterial communities. BMC Microbiol..

[34] Zhang R., Chen L., Cao L., Li K.-J., Huang Y., Luan X.-Q., Li G. (2018). Effects of smoking on the lower respiratory tract microbiome in mice. Respir. Res..

[35] Gu S., Chen D., Zhang J.-N., Lv X., Wang K., Duan L.-P., Nie Y., Wu X.-L. (2013). Bacterial Community Mapping of the Mouse Gastrointestinal Tract. PLoS ONE.

[36] Yatsunenko T., Rey F.E., Manary M.J., Trehan I., Dominguez-Bello M.G., Contreras M., Magris M., Hidalgo G., Baldassano R.N., Anokhin A.P. (2012). Human gut microbiome viewed across age and geography. Nature.

[37] Rinninella E., Raoul P., Cintoni M., Franceschi F., Miggiano G.A.D., Gasbarrini A., Mele M.C. (2019). What is the Healthy Gut Microbiota Composition? A Changing Ecosystem across Age, Environment, Diet, and Diseases. Microorganisms.

[38] Broadley S.P., Plaumann A., Coletti R., Lehmann C., Wanisch A., Seidlmeier A., Esser K., Luo S., Rämer P.C., Massberg S. (2016). Dual-Track Clearance of Circulating Bacteria Balances Rapid Restoration of Blood Sterility with Induction of Adaptive Immunity. Cell Host Microbe.

[39] Zeng Z., Surewaard B.G., Wong C., Geoghegan J.A., Jenne C.N., Kubes P. (2016). CRIg Functions as a Macrophage Pattern Recognition Receptor to Directly Bind and Capture Blood-Borne Gram-Positive Bacteria. Cell Host Microbe.

[40] Iebba V., Guerrieri F., Di Gregorio V., Levrero M., Gagliardi A., Santangelo F., Sobolev A.P., Circi S., Giannelli V., Mannina L. (2018). Combining amplicon sequencing and metabolomics in cirrhotic patients highlights distinctive microbiota features involved in bacterial translocation, systemic inflammation and hepatic encephalopathy. Sci. Rep..

[41] Suppli M.P., Bagger J.I., Lelouvier B., Broha A., Demant M., Kønig M.J., Strandberg C., Lund A., Vilsbøll T., Knop F.K. (2021). Hepatic microbiome in healthy lean and obese humans. JHEP Rep..

[42] Gollwitzer E.S., Saglani S., Trompette A., Yadava K., Sherburn R., McCoy K.D., Nicod L.P., Lloyd C., Marsland B.J. (2014). Lung microbiota promotes tolerance to allergens in neonates via PD-L1. Nat. Med..

[43] Erb-Downward J.R., Thompson D.L., Han M.K., Freeman C.M., McCloskey L., Schmidt L.A., Young V.B., Toews G.B., Curtis J.L., Sundaram B. (2011). Analysis of the lung microbiome in the “healthy” smoker and in COPD. PLoS ONE.

[44] Hilty M., Burke C., Pedro H., Cardenas P., Bush A., Bossley C., Davies J., Ervine A., Poulter L., Pachter L. (2010). Disordered microbial communities in asthmatic airways. PLoS ONE.

[45] Pugliese G., Liccardi A., Graziadio C., Barrea L., Muscogiuri G., Colao A. (2022). Obesity and infectious diseases: Pathophysiology and epidemiology of a double pandemic condition. Int. J. Obes..

[46] González-Muniesa P., Mártinez-González M.-A., Hu F.B., Després J.-P., Matsuzawa Y., Loos R.J.F., Moreno L.A., Bray G.A., Martinez J.A. (2017). Obesity. Nat. Rev. Dis. Prim..

[47] Muscogiuri G., Cantone E., Cassarano S., Tuccinardi D., Barrea L., Savastano S., Colao A. (2019). Gut microbiota: A new path to treat obesity. Int. J. Obes. Suppl..

[48] Turnbaugh P.J., Ley R.E., Mahowald M.A., Magrini V., Mardis E.R., Gordon J.I. (2006). An obesity-associated gut microbiome with increased capacity for energy harvest. Nature.

[49] Binyamin D., Werbner N., Nuriel-Ohayon M., Uzan A., Mor H., Abbas A., Ziv O., Teperino R., Gutman R., Koren O. (2020). The aging mouse microbiome has obesogenic characteristics. Genome Med..

[50] Ley R.E., Turnbaugh P.J., Klein S., Gordon J.I. (2006). Human gut microbes associated with obesity. Nature.

[51] Ley R.E., Bäckhed F., Turnbaugh P., Lozupone C.A., Knight R.D., Gordon J.I. (2005). Obesity alters gut microbial ecology. Proc. Natl. Acad. Sci. USA.

[52] Wang R., Tang R., Li B., Ma X., Schnabl B., Tilg H. (2021). Gut microbiome, liver immunology, and liver diseases. Cell. Mol. Immunol..

[53] Mathieu E., Escribano-Vazquez U., Descamps D., Cherbuy C., Langella P., Riffault S., Remot A., Thomas M. (2018). Paradigms of Lung Microbiota Functions in Health and Disease, Particularly, in Asthma. Front. Physiol..

[54] Befus M., Lowy F.D., Miko B.A., Mukherjee D.V., Herzig C.T.A., Larson E.L. (2015). Obesity as a Determinant of Staphylococcus aureus Colonization Among Inmates in Maximum-Security Prisons in New York State. Am. J. Epidemiol..

[55] Peters U., Dixon A.E., Forno E. (2018). Obesity and asthma. J. Allergy Clin. Immunol..

[56] Litvin M., Yoon J.C., Casella J.L., Blackman S.M., Brennan A.L. (2019). Energy balance and obesity in individuals with cystic fibrosis. J. Cyst. Fibros..

[57] Invernizzi R., Wu B., Barnett J., Ghai P., Kingston S., Hewitt R.J., Feary J., Li Y., Chua F., Wu Z. (2021). The Respiratory Microbiome in Chronic Hypersensitivity Pneumonitis Is Distinct from That of Idiopathic Pulmonary Fibrosis. Am. J. Respir. Crit. Care Med..

[58] Han M.K., Zhou Y., Murray S., Tayob N., Noth I., Lama V.N., Moore B.B., White E.S., Flaherty K.R., Huffnagle G.B. (2014). Lung microbiome and disease progression in idiopathic pulmonary fibrosis: An analysis of the COMET study. Lancet Respir. Med..

[59] D’Alessandro-Gabazza C.N., Kobayashi T., Yasuma T., Toda M., Kim H., Fujimoto H., Hataji O., Takeshita A., Nishihama K., Okano T. (2020). A Staphylococcus pro-apoptotic peptide induces acute exacerbation of pulmonary fibrosis. Nat. Commun..

[60] Fraser J.D., Proft T. (2008). The bacterial superantigen and superantigen-like proteins. Immunol. Rev..

[61] Vu B.G., Stach C.S., Kulhankova K., Salgado-Pabon W., Klingelhutz A.J., Schlievert P.M. (2015). Chronic Superantigen Exposure Induces Systemic Inflammation, Elevated Bloodstream Endotoxin, and Abnormal Glucose Tolerance in Rabbits: Possible Role in Diabetes. mBio.

[62] Vu B.G., Gourronc F.A., Bernlohr D.A., Schlievert P.M., Klingelhutz A.J. (2013). Staphylococcal Superantigens Stimulate Immortalized Human Adipocytes to Produce Chemokines. PLoS ONE.

[63] Shinbori T., Matsuki M., Suga M., Kakimoto K., Ando M. (1996). Induction of Interstitial Pneumonia in Autoimmune Mice by Intratracheal Administration of Superantigen Staphylococcal Enterotoxin B. Cell. Immunol..

