# Supplementary material for: Obesity Reshapes the Microbial Population Structure along the Gut-Liver-Lung Axis in Mice
Source: Biomedicines. 2022 Feb 19;10(2):494. doi: 10.3390/biomedicines10020494 (PMC8962327; doi:10.3390/biomedicines10020494)
Supplement: Supplementary file 1 [file biomedicines-10-00494-s001.zip › biomedicines-1563290-supplementary.pdf]

# Obesity reshapes the microbial population structure along the gut-liver-lung axis in mice

*Apostolos Galaris[1,§], Dionysios Fanidis[1,§], Elli-Anna Stylianaki[1], Vaggelis Harokopos[2], Alexandra-Styliani Kalantzi[1], Panagiotis Moulos[2], Antigone S. Dimas[1], Pantelis Hatzis[2], and Vassilis Aidinis[1,*]*

*[1]Institute of Bioinnovation and [2]Institute for Fundamental Biomedical Research, Biomedical Sciences Research Center Alexander Fleming, 16672 Athens, Greece*

*[§]Equal contribution*
*[*]Correspondence: V.Aidinis@Fleming.gr*

## SUPPLEMENTARY DATA

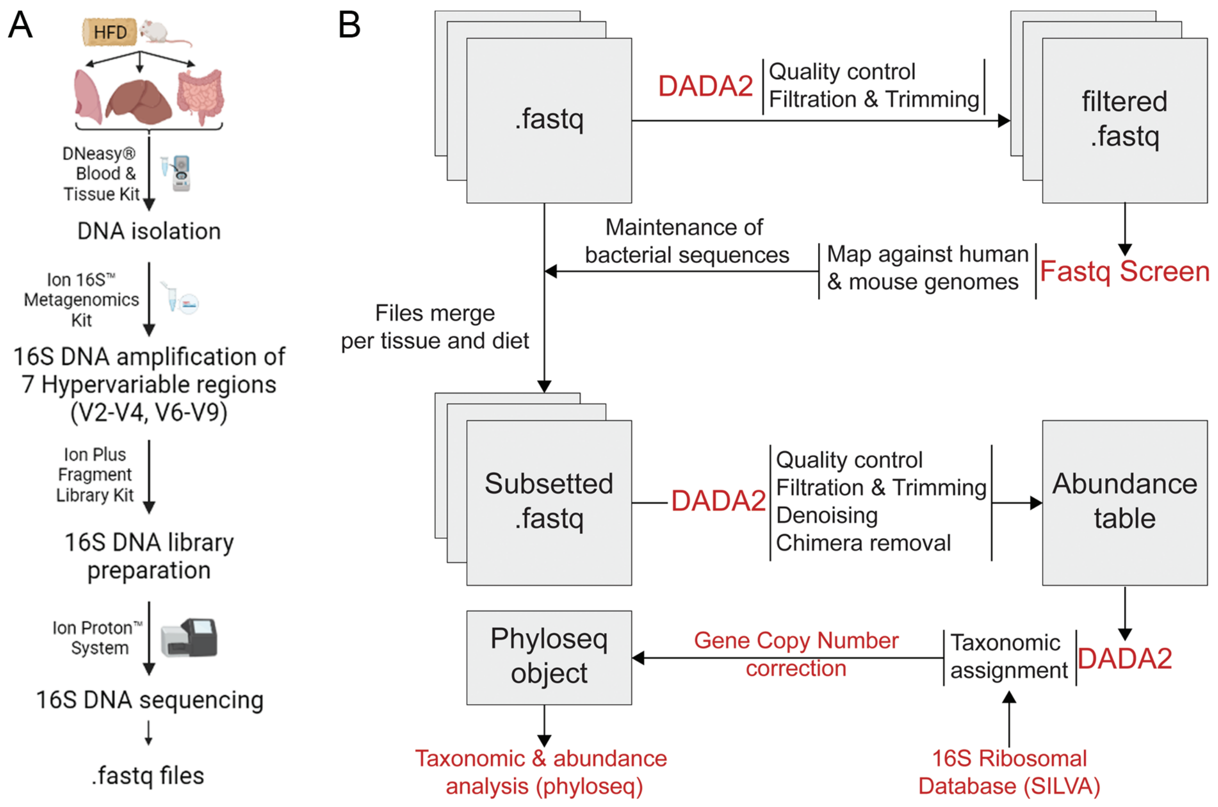


**Figure S1. Schematic overview of experimental design and 16S rDNA sequencing and data analysis.** **(A)** Mice fed with HFD and their relative controls were sacrificed 16 weeks after HFD consumption and their lung, liver, and gut tissues where isolated. Total genomic DNA was extracted, followed by the amplification of seven (V2-V4 and V6-V9) 16S hypervariable regions. 16S DNA libraries were then constructed and sequenced using the Ion Proton™ System. **(B)** FastQ data exported from the Ion Proton™ System were filtered and trimmed using DADA2 for quality control of the reads. Then, abundance tables were constructed for every sample and taxonomy assignment was performed using the SILVA database, followed by Gene Copy Number (GCN) correction. Phyloseq was then used to perform taxonomic and abundance analysis of all samples.

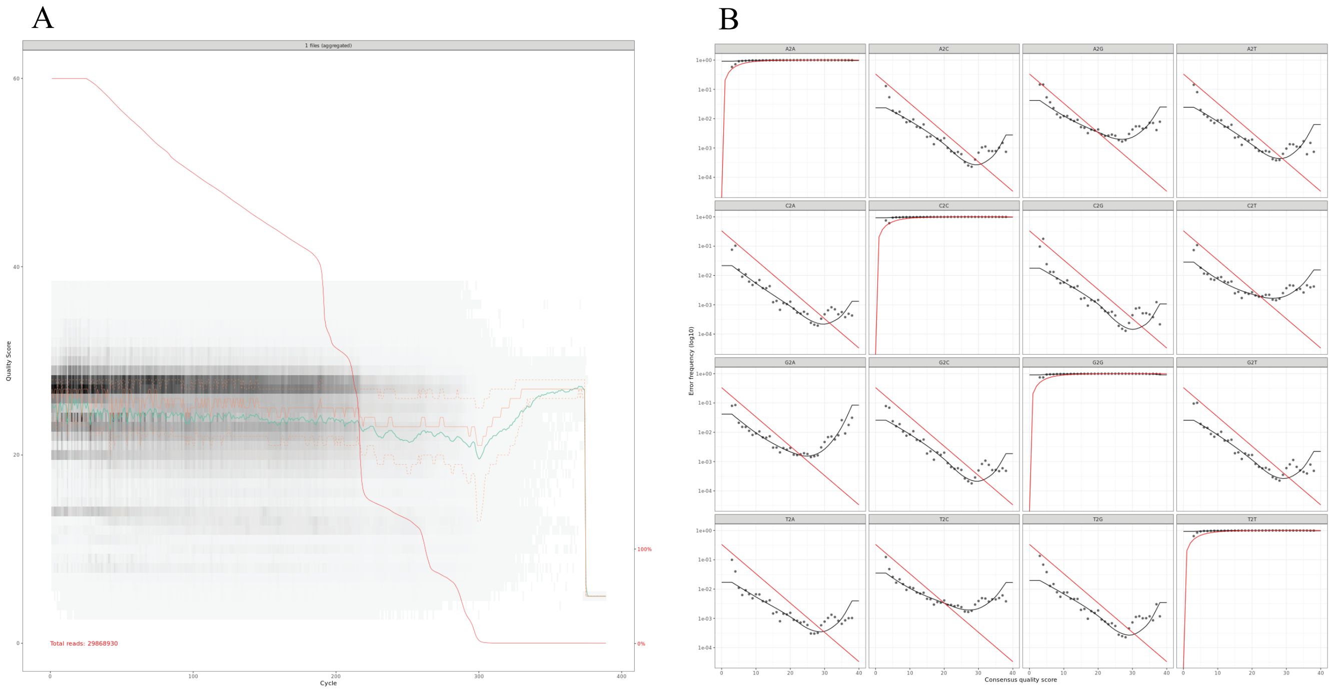


**Figure S2. Data quality control and error rate estimation. A. Read quality profile inspection.** The dataset contains good quality reads as can be seen by the heatmap, the mean (green line) and the quartiles (orange lines) of quality score at each position. **B. Error rates for each nucleotide transition.** The estimated error rates (black line) are a good fit to the observed ones (points). In addition, it can be seen that generally error rates decrease with quality increase, as expected.

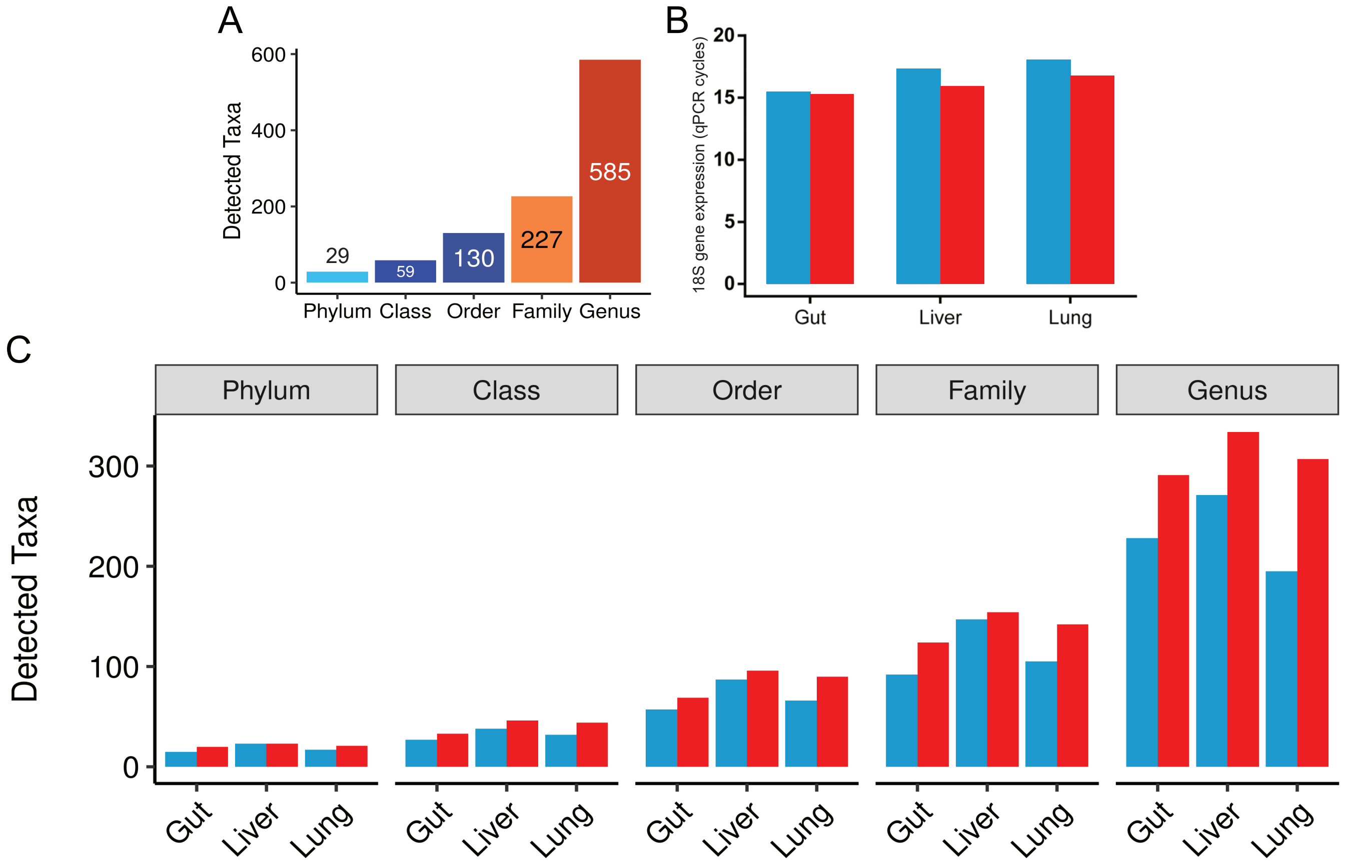


**Figure S3. Obesity increased microbial complexity along the gut-liver-lung axis.** (A) Overall phyla, classes, orders, families, and genera identified. (B) No differences were detected in host tissue 18S rRNA gene, as detected with Q-PCR, ensuring equal sample loading. (C) (HFD)-driven obesity resulted in a tendency for greater number of taxa in all tissues, as compared to the control diet ones.

**Table S1.** Quality control metrics before (grey colored cells) and after (white colored cells) human and bacteria sequences removal.

| Fastq File | Total Input | QC Filtered | Denoised | Chimeras Filtered | #Bacteria | %Bacteria | #Human | %Human | #Mouse | %Mouse |
|---|---|---|---|---|---|---|---|---|---|---|
| GutCtrl_3_2 | 1770986 | 1532202 | 1512880 | 1268758 | 1248402 | 98.4% | 8726 | 0.6% | 27105 | 1.8% |
| | 1731525 | 1498021 | 1486729 | 1246433 | 1246408 | 100.0% | 536 | 0.0% | 810 | 0.1% |
| GutCtrl_3_3 | 1760510 | 1521842 | 1499042 | 1265975 | 1240755 | 98.0% | 7354 | 0.5% | 32938 | 2.2% |
| | 1715354 | 1482715 | 1470362 | 1233805 | 1233611 | 100.0% | 406 | 0.0% | 689 | 0.0% |
| GutHFD_3_1 | 1928020 | 1574046 | 1555039 | 1320228 | 1305339 | 98.9% | 7063 | 0.4% | 17912 | 1.1% |
| | 1900932 | 1550501 | 1537875 | 1309130 | 1306946 | 99.8% | 572 | 0.0% | 900 | 0.1% |
| GutHFD_3_2 | 1948602 | 1609327 | 1589515 | 1363872 | 1353998 | 99.3% | 6560 | 0.4% | 10526 | 0.7% |
| | 1929311 | 1593182 | 1578588 | 1335353 | 1331687 | 99.7% | 516 | 0.0% | 784 | 0.0% |
| GutHFD_3_3 | 1673227 | 1433420 | 1415385 | 1149494 | 1123049 | 97.7% | 6110 | 0.4% | 32992 | 2.3% |
| | 1631187 | 1395532 | 1383614 | 1112892 | 1112289 | 99.9% | 452 | 0.0% | 722 | 0.1% |
| GutHFD_3_4 | 534150 | 461772 | 432985 | 369036 | 326957 | 88.6% | 13178 | 2.9% | 55207 | 12.0% |
| | 455485 | 395226 | 387076 | 320836 | 320056 | 99.8% | 110 | 0.0% | 184 | 0.0% |
| LiverCtrl_3_1 | 374935 | 336859 | 326398 | 266703 | 241140 | 90.4% | 5196 | 1.5% | 30444 | 9.0% |
| | 337707 | 302906 | 299886 | 240129 | 239941 | 99.9% | 1 | 0.0% | 2 | 0.0% |
| LiverCtrl_3_2 | 1679307 | 1531637 | 1498578 | 1390006 | 511884 | 36.8% | 46344 | 3.0% | 916165 | 59.8% |
| | 687747 | 604892 | 597515 | 512209 | 511013 | 99.8% | 1 | 0.0% | 31 | 0.0% |
| LiverCtrl_3_4 | 1718143 | 1315061 | 1294719 | 1130788 | 1104193 | 97.6% | 6150 | 0.5% | 33244 | 2.5% |
| LiverCtrl_3_4 | 1674692 | 1276969 | 1263353 | 1100935 | 1099857 | 99.9% | 690 | 0.1% | 1009 | 0.1% |
| LiverHFD_3_1 | 2547259 | 2232461 | 2212419 | 1816707 | 1803158 | 99.3% | 7725 | 0.3% | 16971 | 0.8% |
| | 2519085 | 2208565 | 2193784 | 1790959 | 1790814 | 100.0% | 626 | 0.0% | 944 | 0.0% |
| LiverHFD_3_2 | 938898 | 789124 | 746957 | 633620 | 594901 | 93.9% | 42609 | 5.4% | 35647 | 4.5% |
| | 852138 | 712640 | 703984 | 589527 | 589376 | 100.0% | 253 | 0.0% | 454 | 0.1% |
| LiverHFD_3_3 | 778814 | 666642 | 650869 | 550628 | 516459 | 93.8% | 6037 | 0.9% | 40773 | 6.1% |
| | 727892 | 621013 | 613072 | 514674 | 514407 | 99.9% | 220 | 0.0% | 385 | 0.1% |
| LiverHFD_3_4 | 912159 | 803836 | 788019 | 659803 | 592211 | 89.8% | 5933 | 0.7% | 77285 | 9.6% |
| | 823363 | 722352 | 714886 | 589288 | 589183 | 100.0% | 243 | 0.0% | 356 | 0.0% |
| LungCtrl_3_1 | 2767079 | 2259528 | 2249469 | 1911019 | 1907913 | 99.8% | 3884 | 0.2% | 6519 | 0.3% |
| | 2755550 | 2250366 | 2240080 | 1906006 | 1902901 | 99.8% | 856 | 0.0% | 1336 | 0.1% |
| LungCtrl_3_2 | 1107349 | 843096 | 826303 | 648015 | 601917 | 92.9% | 4182 | 0.5% | 52427 | 6.2% |
| | 1041219 | 786589 | 773609 | 600137 | 599592 | 99.9% | 443 | 0.1% | 673 | 0.1% |
| LungCtrl_3_3 | 1265273 | 1002180 | 996025 | 858122 | 856185 | 99.8% | 2348 | 0.2% | 1746 | 0.2% |
| | 1260665 | 998144 | 994103 | 855733 | 855568 | 100.0% | 0 | 0.0% | 0 | 0.0% |
| LungHFD_3_1 | 1678219 | 1470977 | 1458803 | 1151079 | 1075140 | 93.4% | 8572 | 0.6% | 80815 | 5.5% |
| | 1584575 | 1384374 | 1379440 | 1075217 | 1075037 | 100.0% | 0 | 0.0% | 1 | 0.0% |
| LungHFD_3_2 | 2271075 | 1831748 | 1811799 | 1557239 | 1420279 | 91.2% | 8140 | 0.4% | 149511 | 8.2% |
| | 2100318 | 1676383 | 1663373 | 1424845 | 1423374 | 99.9% | 704 | 0.0% | 1132 | 0.1% |
| LungHFD_3_3 | 1946944 | 1704019 | 1691277 | 1424228 | 1395615 | 98.0% | 3686 | 0.2% | 32899 | 1.9% |
| | 1907175 | 1667881 | 1657191 | 1384509 | 1383929 | 100.0% | 491 | 0.0% | 827 | 0.0% |
| LungHFD_3_4 | 2294588 | 1969212 | 1953321 | 1657752 | 1613357 | 97.3% | 5564 | 0.3% | 50680 | 2.6% |
| | 2233010 | 1913974 | 1902153 | 1600244 | 1599679 | 100.0% | 592 | 0.0% | 965 | 0.1% |

**Table S2.** Important taxa as presented in Figure 2D, E.

| Inter-tissue common taxa unique per dietary regime | | |
|---|---|---|
| **Taxonomic level** | **Unique in** | **Unique taxa** |
| Phylum | HFD | Fusobacteria; Acidobacteria |
| | Ctrl | - |
| Family | HFD | Peptostreptococcaceae; Muribaculaceae; Rhodobacteraceae; Bacillaceae; Bacteroidaceae; Bifidobacteriaceae; Azospirillaceae; Family_XIII; Listeriaceae; Leuconostocaceae; Flavobacteriaceae; Beijerinckiaceae; Nocardiaceae; Dermacoccaceae; Mitochondria; Sphingobacteriaceae; Nocardioidaceae |
| | Ctrl | - |
| **Inter-tissue common taxa** | | |
| **Taxonomic level** | **Found in** | **Taxa** |
| Phylum | HFD | Bacteroidetes; Proteobacteria; Firmicutes; Actinobacteria; Cyanobacteria |
| | Ctrl | Firmicutes; Actinobacteria; Proteobacteria; Bacteroidetes; Cyanobacteria; Fusobacteria; Acidobacteria |
| Family | HFD | Propionibacteriaceae; Staphylococcaceae; Streptococcaceae; Xanthobacteraceae; Peptoniphilaceae; Corynebacteriaceae; Lactobacillaceae; Micrococcaceae; Pasteurellaceae; Caulobacteraceae; Moraxellaceae; Peptostreptococcaceae; Rhizobiaceae; Prevotellaceae; Lachnospiraceae; Carnobacteriaceae; Neisseriaceae; Actinomycetaceae; Muribaculaceae; Veillonellaceae; Porphyromonadaceae; Burkholderiaceae; Weeksellaceae; Aeromonadaceae; Rhodobacteraceae; Erysipelotrichaceae; Enterobacteriaceae; Ruminococcaceae; Atopobiaceae; Aerococcaceae; Bacillaceae; Fusobacteriaceae; Sphingomonadaceae; Microbacteriaceae; Bacteroidaceae; Bifidobacteriaceae; Azospirillaceae; Pseudomonadaceae; Family_XIII; Listeriaceae; Xanthomonadaceae; Leuconostocaceae; Flavobacteriaceae; Beijerinckiaceae; Nocardiaceae; Dermacoccaceae; Mitochondria; Sphingobacteriaceae; Nocardioidaceae |
| | Ctrl | Xanthobacteraceae; Rhizobiaceae; Caulobacteraceae; Staphylococcaceae; Propionibacteriaceae; Streptococcaceae; Peptoniphilaceae; Erysipelotrichaceae; Corynebacteriaceae; Lactobacillaceae; Burkholderiaceae; Pasteurellaceae; Micrococcaceae; Carnobacteriaceae; Lachnospiraceae; Prevotellaceae; Neisseriaceae; Enterobacteriaceae; Moraxellaceae; Porphyromonadaceae; Veillonellaceae; Atopobiaceae; Actinomycetaceae; Weeksellaceae; Ruminococcaceae; Sphingomonadaceae; Microbacteriaceae; Xanthomonadaceae; Aerococcaceae; Pseudomonadaceae; Aeromonadaceae; Fusobacteriaceae |

**Table S3.** Number of shared taxa between any pairwise combination of experimental conditions.

| | | GutCtrl | GutHFD | LiverCtrl | LiverHFD | LungCtrl | LungHFD |
|---|---|---|---|---|---|---|---|
| Phylum | GutCtrl | - | 5 | 5 | 5 | 5 | 5 |
| | GutHFD | 5 | - | 7 | 7 | 6 | 8 |
| | LiverCtrl | 5 | 7 | - | 7 | 6 | 7 |
| | LiverHFD | 5 | 7 | 7 | - | 6 | 8 |
| | LungCtrl | 5 | 6 | 6 | 6 | - | 6 |
| | LungHFD | 5 | 8 | 7 | 8 | 6 | - |
| | | | | | | | |
| Family | GutCtrl | - | 41 | 34 | 39 | 34 | 36 |
| | GutHFD | 41 | - | 45 | 52 | 38 | 50 |
| | LiverCtrl | 34 | 45 | - | 53 | 40 | 55 |
| | LiverHFD | 39 | 52 | 53 | - | 41 | 59 |
| | LungCtrl | 34 | 38 | 40 | 41 | - | 43 |
| | LungHFD | 36 | 50 | 55 | 59 | 43 | - |

**Table S4.** Detected genera belonging to the Streptococcaceae, Staphylococcaceae, Peptoniphilaceae and Pasteurelacceae families. Marked with red are those that are affected by high fat diet in at least one tissue as defined by HFD-to-control relative abundance difference (non-zero difference).

| Phylum | Family | Genus |
|---|---|---|
| Firmicutes | Streptococcaceae | Streptococcus |
| | | Lactococcus |
| | Staphylococcaceae | Staphylococcus |
| | | Salinicoccus |
| | | Jeotgalicoccus |
| | Peptoniphilaceae | Gemella |
| | | Peptoniphilus |
| | | Anaerococcus |
| | | Finegoldia |
| | | Gallicola |
| | | Parvimonas |
| | | Tissierella |
| | | Ezakiella |
| | | Murdochiella |
| | | W5053 |
| | | Sedimentibacter |
| Proteobacteria | Pasteurelacceae | NA |

**Table S5.** Detected species related with superantigen proteins as recorded by UniProt database and/or respective literature.

| Phylum | Family | Species | Source |
|---|---|---|---|
| Firmicutes | Peptoniphilaceae | *Finegoldia magna* | Literature |
| | Streptococcaceae | *Lactobacillus delbruecki* | TrEBML |
| | | *Lactococcus lactis* | TrEBML |
| | Staphylococcaceae | *Staphylococcus aureus* | KB; TrEBML; Literature |
| | | *Staphylococcus massiliensis* | TrEBML |
| | | *Staphylococcus epidermis* | TrEBML |
| Proteobacteria | Moraxellaceae | *Acinetobacter baumannii* | TrEBML |
